# Reinforcement Learning for Clinical Decision Support in Critical Care: Comprehensive Review

**DOI:** 10.2196/18477

**Published:** 2020-07-20

**Authors:** Siqi Liu, Kay Choong See, Kee Yuan Ngiam, Leo Anthony Celi, Xingzhi Sun, Mengling Feng

**Affiliations:** 1 NUS Graduate School for Integrative Science and Engineering National University of Singapore Singapore Singapore; 2 Saw Swee Hock School of Public Health National University of Singapore Singapore Singapore; 3 Division of Respiratory & Critical Care Medicine National University Hospital Singapore Singapore; 4 Group Chief Technology Office National University Health System Singapore Singapore; 5 Institute for Medical Engineering and Science Massachusetts Institute of Technology Cambridge, MA United States; 6 Division of Pulmonary, Critical Care and Sleep Medicine Beth Israel Deaconess Medical Center Boston, MA United States; 7 Ping An Health Technology Beijing China

**Keywords:** artificial intelligence, reinforcement learning, critical care, decision support systems, clinical, intensive care unit, machine learning

## Abstract

**Background:**

Decision support systems based on reinforcement learning (RL) have been implemented to facilitate the delivery of personalized care. This paper aimed to provide a comprehensive review of RL applications in the critical care setting.

**Objective:**

This review aimed to survey the literature on RL applications for clinical decision support in critical care and to provide insight into the challenges of applying various RL models.

**Methods:**

We performed an extensive search of the following databases: PubMed, Google Scholar, Institute of Electrical and Electronics Engineers (IEEE), ScienceDirect, Web of Science, Medical Literature Analysis and Retrieval System Online (MEDLINE), and Excerpta Medica Database (EMBASE). Studies published over the past 10 years (2010-2019) that have applied RL for critical care were included.

**Results:**

We included 21 papers and found that RL has been used to optimize the choice of medications, drug dosing, and timing of interventions and to target personalized laboratory values. We further compared and contrasted the design of the RL models and the evaluation metrics for each application.

**Conclusions:**

RL has great potential for enhancing decision making in critical care. Challenges regarding RL system design, evaluation metrics, and model choice exist. More importantly, further work is required to validate RL in authentic clinical environments.

## Introduction

### Background

In the health care domain, clinical processes are dynamic because of the high prevalence of complex diseases and dynamic changes in the clinical conditions of patients. Existing treatment recommendation systems are mainly implemented using rule-based protocols defined by physicians based on evidence-based clinical guidelines or best practices [1-3]. In addition, these protocols and guidelines may not consider multiple comorbid conditions [4]. In an intensive care unit (ICU), critically ill patients may benefit from deviation from established treatment protocols and from personalizing patient care using means not based on rules [5,6].

When physicians need to adapt treatment for individual patients, they may take reference from randomized controlled trials (RCTs), systemic reviews, and meta-analyses. However, RCTs may not be available or definitive for many ICU conditions. Many patients admitted to ICUs might also be too ill for inclusion in clinical trials [6]. Furthermore, only 9% of treatment recommendations in the ICU are based on RCTs [7], and the vast majority of RCTs in critical care have negative findings [8]. To aid clinical decisions in ICUs, we need other methods, including the use of large observational data sets. ICU data can be useful for learning about patients as they were collected in a data-rich environment. A large amount of data can then be fed into artificial intelligence (AI) systems (using computers to mimic human cognitive functions) and machine learning methods (using computer algorithms to perform clinical tasks without the need for explicit instructions). AI and machine learning can then help with diagnosis [9,10], treatment [11,12], and resource management [13,14] in the ICU. Given the dynamic nature of critically ill patients, one machine learning method called reinforcement learning (RL) is particularly suitable for ICU settings.

### Fundamentals of Reinforcement Learning

RL is a goal-oriented learning tool where a computer *agent*, acting as a decision maker, analyzes available data within its defined environment [15], derives a rule for taking actions, and optimizes long-term rewards. The agent is the RL model that we wish to develop. In general, an RL agent receives evaluative feedback about the performance of its action in each time step, allowing it to improve the performance of subsequent actions by trial and error [16]. Mathematically, this sequential decision-making process is called the Markov decision process (MDP) [17]. An MDP is defined by 4 major components: (1) a state that represents the environment at each time; (2) an action the agent takes at each time that influences the next state; (3) a transition probability that provides an estimate for reaching different subsequent states, which reflects the environment for an agent to interact with; (4) a reward function is the observed feedback given a state-action pair. The solution of the MDP is an optimized set of rules and is termed the policy.

RL has already emerged as an effective tool to solve complicated control problems with large-scale, high-dimensional data in some application domains, including video games, board games, and autonomous control [18-20]. In these domains, RL has been proven to achieve human-level capacity for learning complex sequential decisions. For instance, Alpha Go is an RL agent for playing the strategy board game Go. On the basis of Alpha Go’s learned policy, and given the current position of the Go stones, it is possible to decide where the next white/black stone should be placed on the board to maximize its chance of winning.

### Analogies to Critical Care

For critical care, given the large amount and granular nature of recorded data, RL is well suited for providing sequential treatment suggestions, optimizing treatments, and improving outcomes for new ICU patients. RL also has the potential to expand our understanding of existing clinical protocols by automatically exploring various treatment options. The RL agent analyzes the patient trajectories, and through trial and error, derives a policy, a personalized treatment protocol that optimizes the probability of favorable clinical outcomes (eg, survival). As this computerized process is an attempt to mimic the human clinician’s thought process, RL has also been called the AI clinician [21].

We can consider the state as the well-being/condition of a patient. The state of the patients could depend on static traits (eg, patient demographics including age, gender, ethnicity, pre-existing comorbidity) and longitudinal measurements (eg, vital signs, laboratory test results). An action is a treatment or an intervention that physicians do for patients (eg, prescription of medications and ordering of laboratory tests). The transition probability is the likelihood of state transitions, and it is viewed as a prognosis. If the well-being in the new state is improved, we assign a reward to the RL agent, but we penalize the agent if the patient's condition worsens or stays stagnant after the intervention.

**Figure 1:**
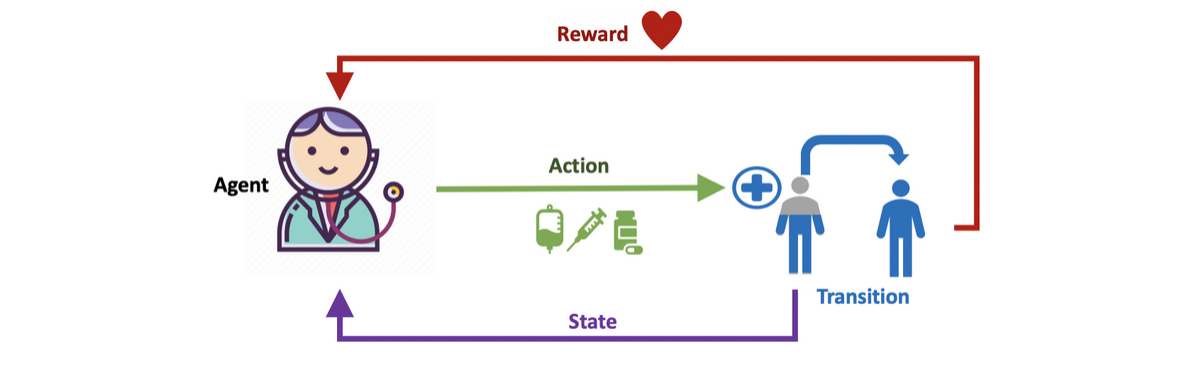
Illustration of reinforcement learning in critical care.

As illustrated in Figure 1, if we take a snapshot of the current well-being of a patient as his/her state, the physician would provide a treatment or an intervention (an action) to the patient. This action would lead the patient to the next state depending on his/her current state and the action performed on him/her. While knowing the next state of the patient, the physician would need to take another action according to the new state. These state-action pairs would continue to rollout over time, and the resultant trajectory of state-action pairs could represent the changes in the patients’ conditions and the sequential treatment decisions that were performed by the physicians. We can define the length of the trajectory for each patient as fixed (eg, during the first 24 hours of the ICUs stay) or as dynamic (eg, different patients could be discharged from the ICUs at different times).

The main objective of the RL algorithm is to train an agent that can maximize the cumulative future reward from the state-action pairs given the patients’ state-action trajectories. When a new state is observed, the agent is able to perform an action, which could choose the action for the greatest long-term outcome (eg, survival). When the RL agent is well-trained, it is possible to pick the best action given the state of a patient, and we describe this process as acting according to an optimal policy.

A policy is analogous to a clinical protocol. Nonetheless, a policy has advantages over a clinical protocol because it is capable of capturing more personalized details of individual patients. A policy can be represented by a table where it maps all possible states with actions. Alternatively, a policy could also be represented by a deep neural network (DNN) where given the input of a patient’s state, the DNN model outputs the highest probability of an action. An optimal policy can be trained using various RL algorithms. Some widely applied RL algorithms include the fitted-Q-iteration (FQI) [22], deep Q network (DQN) [23], actor-critic network [24], and model-based RL [25]. More technical details about various RL models have been explained [26,27].

As RL in critical care is a relatively nascent field, we therefore aimed to review all the existing clinical applications that applied RL in the ICU setting for decision support over the past 10 years (2010-2019). Specifically, we aimed to categorize RL applications and summarize and compare different RL designs. We hope that our overview of RL applications in critical care can help reveal both the advances and gaps for future clinical development of RL. A detailed explanation of the concept of RL and its algorithms is available in Multimedia Appendix 1 [28].

## Methods

### Search Strategy

A review of the literature was conducted using the following 7 databases: PubMed, Institute of Electrical and Electronics Engineers (IEEE), Google Scholar, Medical Literature Analysis and Retrieval System Online (MEDLINE), Excerpta Medica Database (EMBASE), ScienceDirect, and Web of Science. The search terms *reinforcement learning, critical care, intensive care, intensive care units,* and *ICUs* were combined. The search phrases listed in Textbox 1 were used to identify articles in each database.

**Queries used to retrieve records.**

EMBASE (Excerpta Medica Database)

- #1 ‘reinforcement learning’
- #2 ‘intensive care unit’ OR ‘critical care’ OR ‘ICU’
- #1 AND #2

Google Scholar

- (conference OR journal) AND (“intensive care unit” OR “critical care” OR ICU) AND “reinforcement learning” -survey -reviews -reviewed -news

IEEE (Institute of Electrical and Electronics Engineers)

- ((“Full Text Only”: “reinforcement learning”) AND “Full Text Only”: “intensive care units”) OR ((“Full Text Only”: “reinforcement learning”) AND “Full Text Only”: “critical care”)

MEDLINE (Medical Literature Analysis and Retrieval System Online)

- multifield search=reinforcement learning, critical care, intensive care

PubMed

- (“reinforcement learning”) AND ((“ICU”) OR (“critical care”) OR (“intensive care unit”) OR (“intensive care”))

ScienceDirect

- “reinforcement learning” AND (“critical care” OR “intensive care” OR “ICU”)

Web of Science

- ALL=(intensive care unit OR “critical care” OR “ICU”) AND ((ALL=(“reinforcement learning”)) AND LANGUAGE: (English))

### Inclusion Criteria

To be eligible for inclusion in this review, the primary requirement was that the article needed to focus on the implementation, evaluation, or use of an RL algorithm to process or analyze patient information (including simulated data) in an ICU setting. Papers published from January 1, 2010, to October 19, 2019 were selected. General review articles and articles not published in English were excluded. Only papers that discussed sufficient details on the data, method, and results were included in this review.

### Data Synthesis

Data were manually extracted from the articles included in the review. A formal quality assessment was not conducted, as relevant reporting standards have not been established for articles on RL. Instead, we extracted the following characteristics from each study: the purpose of the study, data source, number of patients included, main method, evaluation metrics, and related outcomes. The final collection of articles was divided into categories to assist reading according to their application type in the ICUs.

## Results

### Selection Process and Results Overview

**Figure 2:**
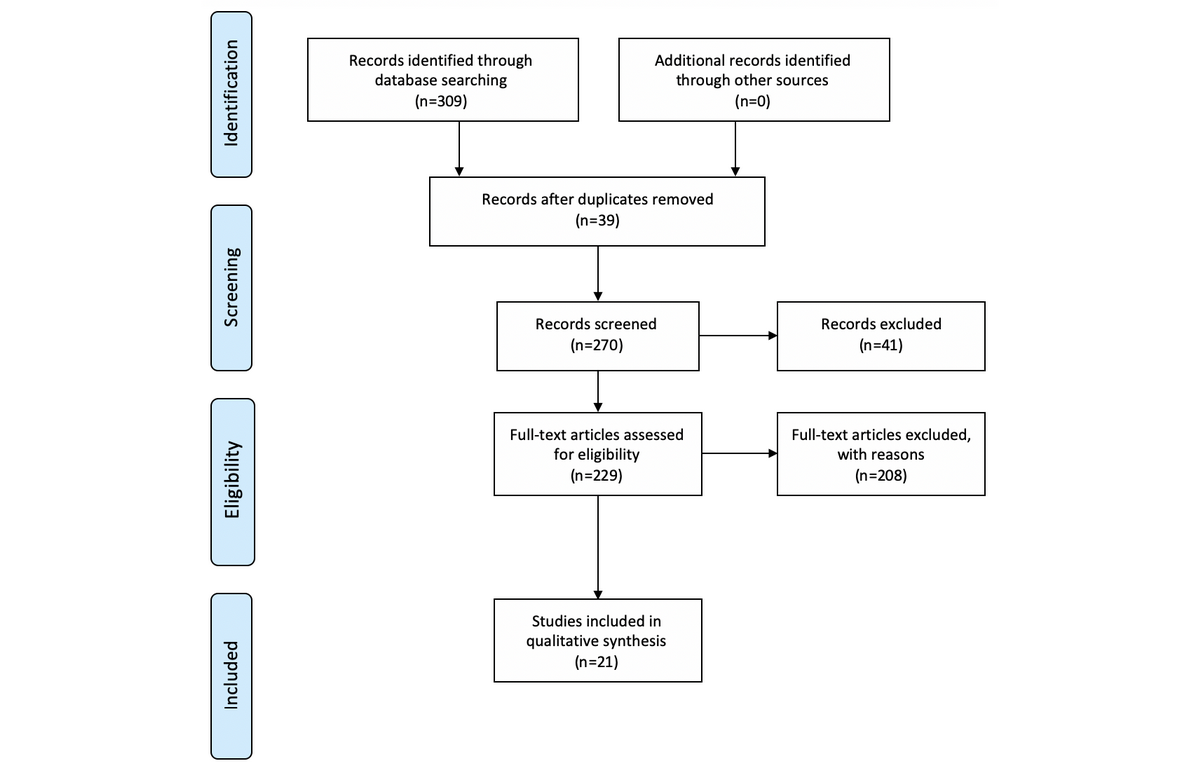
Preferred Reporting Items for Systematic Reviews and Meta-Analyses flow diagram of the search strategy.

The selection process of this review was demonstrated using the Preferred Reporting Items for Systematic Reviews and Meta-Analyses flow diagram (Figure 2). From the full text of 269 distinct articles, an independent assessment for eligibility was performed by 2 authors (SL and MF). Disagreements were discussed to reach consensus. During the full-text review, 249 articles were excluded, and 21 articles were eventually included. The reasons for exclusion during the review process are outlined in Table 1.

**Table 1:** Exclusion criteria used to exclude papers.

Criterion number	Exclusion criteria	Justification	Excluded articles, n
1	Duplicates	The papers have duplicate titles	39
2	Not a research article	The papers were blog articles, reports, comments, or views	23
3	Not written in English	The papers were not written in English	6
4	Review	The papers were review articles regarding general methods on big data, deep learning, and clinical applications	12
5	Not applied in the field of critical care	The papers did not focus on applications in critical care or intensive care	92
6	Not using RL^a^ as the approach in critical care	The papers discussed issues in the critical care setting, but not using RL as an approach	115
7	No clear description of the method and result	The methods and results were not clearly described and thus not qualified for this review	1

^a^RL: reinforcement learning.

In this section, we organized the reviewed articles into 4 categories, which reflect clinically relevant domains: (1) optimal individualized target laboratory value; (2) optimal choice of medication; (3) optimal timing of an intervention; and (4) optimal dosing of medication.

**Figure 3:**
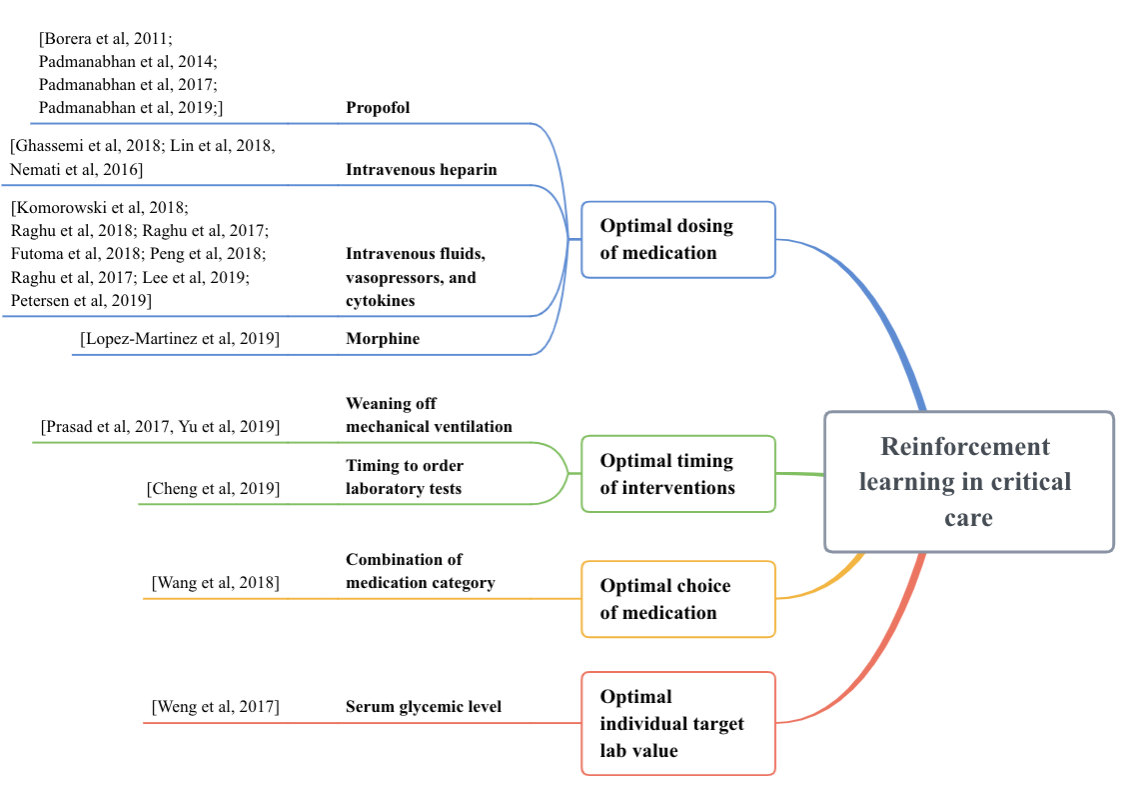
Mapping of reinforcement learning studies in critical care by application type.

We plotted the number of articles reviewed by their category and year of publication in Figure 3. We found that the majority of the papers were published in the past 3 years (n=17), indicating an increasing trend of applying RL-based approaches to assist physicians in decision making in critical care. In each of the 4 categories, we further organized the articles into subgroups based on their clinical questions (Figure 3). The figure shows that most of the applications used RL to find optimal drug dosing (n=16) [6,21,29-42], followed by the timing of an intervention (n=3) [43-45]. Only a few applications were looking at the individualized laboratory value (n=1) [46] and the optimal choice of medication (n=1) [47].

Next, we discuss the details for each category with the methods and outcomes for each application. In particular, we further grouped the studies based on specific medication or treatment type in categories 3 and 4 to assist readers. A summary of all study details is found in Multimedia Appendix 2.

### Optimal Individualized Target Laboratory Value

Even after decades of routine use of laboratory value ranges, reference standards may need to be reconsidered, especially for individual patients [48]. Personalized targets for laboratory values in ICU patients could account for disease severity, comorbidities, and other patient-specific differences. Weng et al [46] tried to identify individualized targeted blood glucose levels as a reference for physicians. They applied an RL-based approach, *policy iteration*, to learn the target glycemic range at an hourly interval for severely ill patients with sepsis using real ICU data. Their approach was tested using the Medical Information Mart for Intensive Care III (MIMIC III), a large, publicly available ICU database [49]. MIMIC III contains information for hospital admissions of 43,000 patients in critical care units during 2001 and 2012, from which the authors extracted hourly data for 5565 patients with sepsis.

Weng et al [46] constructed their RL model as follows: First, they represented the patients’ states from 128 variables. These variables included patient demographics, comorbid conditions, vital sign changes, and laboratory value changes. They used a spare autoencoder [50] to reduce the high dimensionality of the raw features (128 dimensions) to only 32 dimensions so that the RL model could be trained more efficiently with limited observational data. Second, they chose to act upon 1 of 11 discrete ranges of serum glucose at each time step. Third, they designed the reward function so that the RL agent could recommend an hourly target glucose level to optimize long-term survival. A positive 100 was assigned to the end state if patients survived 90 days after admission, and a negative 100 was assigned if the patients died. For each state-action pair, the *value* of the pair was iteratively estimated using the reward from the training data.

To understand how the reward value was related to mortality, the authors assigned values to discrete buckets using separate test data. In each value bucket, if the state-action pair is part of a trajectory where a patient died, a label of 1 was assigned to that bucket; otherwise, a label of 0 was assigned. After assigning all the state-action pairs from the test data with the labels in the corresponding value bucket, the mortality rate could be estimated for each value bucket. The authors plotted the estimated mortality rate with respect to the value-buckets and found an inverse relationship between them, where the highest value was associated with the lowest mortality. This result suggested that the learnt value represented the relationship between the state-action pair and mortality and that the learnt value of the state-action pairs from training data was validated on the test data.

To validate the RL policy, the author calculated the frequency of state transitions from the training data and generated new trajectories. Starting from the observed state in the test data, the RL policy would recommend an action with the highest value, and the subsequent state was estimated with the transition probability. By averaging the value for all state-action pairs in the simulated trajectory, the mortality for simulated trajectories could be estimated by mapping this value in the mortality-value plot. Compared with the actual mortality rate in the test data, the author claimed that if physicians could control patients’ hourly blood glucose levels within the range recommended by the RL model, the estimated 90-day mortality would be lowered by 6.3% (from 31% to 24.7%).

### Optimal Choice of Medications

Apart from some clinical decision support systems, commonly used systems such as computerized prescriber order entry and bar-coded medication administration lack personalized recommendations to optimize medication effectiveness and minimize side effects [51]. Wang et al [47] applied a deep learning network based on RL to exploit medication recommendations with a data-driven strategy. Their approach accounted for individual patient demographics, laboratory values, vital signs, and diagnoses from the MIMIC III database. They selected the top 1000 out of 4127 medications and the top 2000 out of 6695 diseases (represented by the International Classification of Diseases, Ninth Revision codes), which covered 85.4% of all medication records and 95.3% of all diagnosis records, respectively. To reduce the problem complexity, the authors further categorized the 1000 medications into 180 drug categories using anatomical therapeutic chemical codes and aggregated patients’ drug prescriptions into 24-hour windows.

The authors defined RL action as the medication combinations from the 180 drug categories. They adopted an actor-critic RL agent that suggested a daily medication prescription set, and aimed to improve patients’ hospital survival. The details of the actor-critic RL algorithm are explained in Multimedia Appendix 1 [28]. For each patient’s ICU day, the *actor* network would recommend one medication combination by considering state variables such as demographics, laboratory results, and vital signs. A reward value of positive 15 would be given to the end state if a patient survived until hospital discharge and negative 15 if the patient died. The reward was designated as 0 for all other time steps. To counterbalance the *actor* network, the *critic* network was applied to evaluate the consistency of actual physician prescriptions and the RL agent’s recommendations. The net effect of the actor-critic RL agent was to optimize the long-term outcomes of patients (hospital mortality) while minimizing deviations of RL-recommended actions from actual prescription patterns. In addition to the actor-critic network, the authors also applied long short-term memory [52] to represent a patient’s current state by incorporating the long sequence of all historical states. Wang et al [47] suggested that hospital mortality would be reduced by 4.4% if clinicians adhered to the RL agent’s recommendations.

### Optimal Timing of Intervention

#### Weaning of Mechanical Ventilation

Mechanical ventilation (MV) is a life-saving treatment applied in approximately a third of all critically ill patients [53]. Prematurely discontinuing MV (premature weaning) and excessively prolonged MV (late weaning) are both associated with higher mortality [54]. The best time to wean may be uncertain [55].

To optimize the timing of ventilation discontinuation, Prasad et al [43] applied the RL-based FQI (the details of the FQI algorithm are explained in Multimedia Appendix 1 [28]) on the MIMIC III database for all patients who were kept under ventilator support for more than 24 hours and extracted their records every 10 min from ICU admission to discharge. Patient states included a number of factors that could affect extubation, such as demographics, pre-existing conditions, comorbidities, and time-varying vital signs. The action for the ventilation setting was binary, that is, for each 10-min time step, the RL agent needed to decide whether the ventilation should be set on (continued MV) or off (weaned from MV). For reward design, Prasad et al [43] followed an existing weaning protocol from the Hospital of University of Pennsylvania. They assigned reward values to the RL agent at each time step according to 3 major considerations: (1) the RL agent should penalize each additional hour spent on the ventilator, (2) the RL agent should be assigned a positive reward value to a weaning action if the patient’s vital signs and laboratory results were steady and within normal ranges after extubation, and (3) there was no reward value for failed spontaneous breathing trial or for reintubation after the first extubation. For RL policy evaluation, the authors calculated the proportion of weaning actions from the RL policy, referencing the total number of weaning actions from the clinician’s policy at each time step, and calculated the overall consistency of weaning transitions. The recommend actions from the RL agent could match 85% of those from clinicians. The authors categorized the degree of consistency into 5 bins, and plotted the distribution of the number of reintubations with respect to the discrete consistency levels. Their results showed that when the consistency was high, vital sign fluctuations were fewer, laboratory results were more in-range, and reintubations were minimized.

Yu et al [45] studied the same clinical issue as Prasad et al [43] and used the same data set, but designed a different reward function using inverse RL. The inverse RL model directly learnt reward mapping from data for each state-action pair and inferred what clinicians would wish to achieve as a reward. Similar to Prasad et al [43], the RL recommendations by Yu et al [45] were associated with shorter weaning times and fewer reintubations compared with clinician decision making.

#### Timing to Order Laboratory Tests

The timing of ordering a laboratory test can be challenging. Delayed testing would lead to continued uncertainty over the patient’s condition and possible late treatment [56]. However, excessively early ordering of laboratory tests can cause unnecessary discomfort to the patient, increase the risk of anemia, and increase health care cost.

Cheng et al [44] applied the FQI method to find the optimal timing for ordering laboratory tests among patients with sepsis in the MIMIC III data set. They examined the timing of 4 types of laboratory tests: white blood cell count (WBC), creatinine, blood urea nitrogen (BUN), and lactate. They sampled the patients’ data at hourly intervals and constructed the state of a patient by considering the predictive variables of severe sepsis or acute kidney failure, including respiratory rate, heart rate, mean blood pressure, temperature, creatinine, BUN, WBC, and lactate. The missing values were predicted by a multioutput Gaussian process [57,58]. In their RL model, they chose to design the reward function with the combination of 4 factors: (1) a positive reward should be given only if the ordering of test was necessary, while penalizing over or under ordering; (2) the RL agent should be encouraged to order laboratory tests when there was a sudden change in laboratory results or vital signs; (3) negative reward should be given if the laboratory results were similar to the last measurements (no information gain); (4) a penalty would be added to a reward whenever a test was ordered, to reflect the testing cost. Their RL agent, compared with clinicians, was able to reduce the number of laboratory tests by 27% for lactate and 44% for WBC, while maintaining high information gain.

### Optimal Dosing of a Drug

Recommendations for dosing regimens in ICU patients are often extrapolated from clinical trials in healthy volunteers or noncritically ill patients. This extrapolation assumes similar drug behavior (pharmacokinetics and pharmacodynamics) in the ICU and other patients or healthy volunteers. However, it is well known that many drugs used in critically ill patients may have alterations in pharmacokinetic and pharmacodynamic properties because of pathophysiological changes or drug interactions [59]. Therefore, critically ill patients bring unique challenges in drug dosing.

#### Dosing of Propofol

Critically ill patients in ICUs often require sedation to facilitate various clinical procedures and to comfort patients during treatment. Propofol is a widely used sedative medication [60], but titration of propofol is challenging, and both over sedation and under sedation can have adverse effects [32]. Of the studies reviewed, 6 studies have focused on applying RL to determine the optimal dosage for propofol while maintaining the physiological stability of the patient. The bispectral index (BIS) was used to monitor sedation level and to determine the effect of propofol.

Borera et al [29] was the first to apply RL to a pharmacokinetic model [61] to describe the time-dependent distribution of propofol in human surgical patients. The RL agent was a neural network aimed at optimizing the propofol dose to achieve the target BIS value. The patient’s state and state transition were modeled using a mathematical pharmacokinetic model with predefined parameters such as the concentration at half maximal effect of BIS, degree of nonlinearity of BIS, and time-lag coefficient to estimate the BIS value for simulated patients. The action was a discrete range of propofol infusion rate. The reward function was the error rate between the target BIS value and the current simulated BIS value, where a larger negative reward was given when the current simulated BIS value was further away from the predefined target value. They measured the performance of the RL agent by looking at the time to reach the target BIS value (steady time). The evaluation was conducted on 1000 simulated patients. On average, the steady time was 3.25 min for the BIS value to reach target.

To ensure patient safety, propofol dosing should consider the concurrent stability of vital parameters. For instance, Padmanabhan et al [30] chose mean arterial pressure (MAP) as the secondary control variable. The authors combined the error rates for both BIS and MAP when designing the reward. The target for the RL agent was to infuse propofol so that the target BIS would be reached in a short time, whereas MAP was kept within a desired range. In subsequent studies, Padmanabhan et al [31,32] modified their methods with different RL training algorithms (Q-learning and policy iteration). In all their studies, the RL agent was able to suggest accurate propofol doses and achieve target BIS values within a few minutes.

In contrast to fixed pharmacokinetic models in the RL model environment, Yu et al [45] applied FQI and Bayesian inverse RL on the MIMIC III database. They considered patients’ demographic characteristics, pre-existing conditions, comorbidities, and time-varying vital signs to construct the state of the patient. Their inverse RL model interpreted clinician preference as a reward for different patient states. The learned reward function from the inverse RL model suggested that clinicians may pay more attention to patients’ cardiorespiratory stability rather than oxygenation when making decisions about propofol dosage.

#### Dosing of Intravenous Heparin

Anticoagulant agents are often used to prevent and treat a wide range of cardiovascular diseases. Heparin is commonly used in critical care [62], yet its precise dosing is complicated by a narrow therapeutic window. Overdosing of heparin results in bleeding whereas under dosing risks clotting. To guide heparin dosing, activated partial thromboplastin time (aPTT) is often used as a measure of the anticoagulant effect of heparin.

Nemati et al [6] applied FQI with a neural network to optimize and individualize heparin dosing. Their study was conducted on the MIMIC II database, with the reward function based on aPTT levels following heparin dosing [63]. The reward to the RL agent will be high if the aPTT value is between 60 and 100 seconds. After training, they plot the state-action value with respect to the level of consistency between the RL policy and clinician practice. Their results showed that, on average, following the recommendations of the RL agent resulted in higher state-action values.

Ghassemi et al [33] and Lin et al [34] focused on a personalized optimal heparin dosing using different RL algorithms. In addition to the MIMIC III data set, Lin et al [34] applied an actor-critic network on the Emory Healthcare data set from Emory University. For RL policy evaluation, Lin et al [34] regressed the discordance between RL policy and physician practice over the number of clotting and bleeding complications, adjusting for covariates such as history of clot or bleed, weight, age, and sequential organ failure assessment score. The regression coefficient suggested that following the RL agent’s recommendations would have likely resulted in improved clinical outcomes with a reduced number of clotting and bleeding complications.

#### Intravenous Fluids, Vasopressors, and Cytokine Therapy for Treating Sepsis

Sepsis is the third leading cause of death and is expensive to treat [64]. Besides antibiotics and source control, challenges remain with the use of intravenous (IV) fluids to correct hypovolemia and administration of vasopressors to counteract sepsis-induced vasodilation. Raghu et al [36] suggested a data-driven RL approach to recommend personalized optimal dosage for IV fluids and vasopressors to improve hospital mortality. Their RL model was double DQN with dueling, which can minimize the overestimation problem of previous Q-learning models. The details of the Q-learning and double DQN algorithms are explained in Multimedia Appendix 1 [28]. The authors considered patients’ demographics, laboratory values, vital signs, and intake/output events as state features in the RL model. Action was designed as a combination of 5 discrete bins for IV fluid dosing and 5 bins for vasopressor dosing to treat patients with sepsis. The reward was issued at the terminal time step of the patient’s trajectory, with a positive reward if the patient survived. Data were extracted from the MIMIC III database for all patients who fulfilled sepsis-3 criteria [65]. For policy evaluation, Raghu et al [36] plotted the estimated hospital mortality with respect to the difference between dosages recommended by the RL agent and by clinicians. The plot showed that the mortality was lowest when there was no discrepancy between RL policy and physician decision making. Six other groups of researchers also focused on the same research question and applied various RL algorithms with slightly different designs of the state space, reward function, and evaluation metrics [21,35,37-40]. The findings from these studies all suggest that the RL agent would be able to learn from the data and if physicians followed the RL policy, the estimated hospital mortality could be improved.

Among the aforementioned studies, Komorowski et al [21] were the pioneers of applying RL in the ICU, using data from patients with sepsis in the MIMIC III database. They inferred a patient’s health status using an array of inputs, which included demographics, vital signs, laboratory tests, illness severity scores, medications, procedures, fluid intake and output, physician notes, and diagnostic coding. Patient data were aggregated and averaged every 4 hours to represent patient states. Using a k-means algorithm, these patient states were then simplified into 750 discrete mutually exclusive clusters. A sequence of these clustered states would describe a particular patient’s trajectory. The authors estimated the state transition probability by counting how many times each transition was observed and converted the counts to a stochastic matrix. This transition matrix contained the probability for each patient going to a new state, given a previous action taken in the current state. The entire trajectory of a patient’s state can be estimated using the transition matrix. The authors applied a policy iteration RL algorithm that learnt the optimal dosing policy for IV fluids and vasopressors to maximize the probability of 90-day survival.

Nevertheless, the study by Komorowski et al [21]. had several limitations. First, their study only considered fluid and vasopressor management, ignoring other important treatments such as source control, correction of hypovolemia, and management of secondary organ failures [21]. Second, 90-day mortality is affected by factors outside of the ICU, which the study did not take into account. Third, clinical decision making considers both short-term outcomes (eg, physiological stability) and long-term outcomes (eg, kidney failure or mortality), but the study only considered mortality as the single goal for training the RL algorithm [66]. Fourth, discretizing patient health status into discrete clusters loses data granularity and may limit the ability to detect changes in patient status. These limitations also occur in other studies, which we will elaborate in the Discussion section.

Other than using IV fluids and vasopressors for treating sepsis. Petersen et al [42] investigated cytokine therapy using the deep deterministic policy gradient [67] method. The details of the policy gradient RL algorithm are explained in Multimedia Appendix 1 [28]. They evaluated the RL model by using an agent-based model, the innate immune response agent-based model [68], that simulated the immune response to infection. The RL policy was able to achieve a very low mortality rate of 0.8% over 500 simulated patients, and suggested that personalized multicytokine treatment could be promising for patients with sepsis.

#### Dosing of Morphine

Critically ill patients may experience pain as a result of disease or certain invasive interventions. Morphine is one of the most commonly used opioids for analgesia [69]. Similar to sedation, the dosing of analgesia is subject to uncertainty. Lopez-Martinez et al [41] collected data for patients who had at least one pain intensity score and at least one dose of IV morphine in the MIMIC III database. They applied double DQN with dueling as their RL model and constructed the state space to be continuous with features including the patient’s self-reported pain intensity and their measured physiological status. The action was a choice of 14 discrete dosing ranges of IV morphine. The reward was determined by considering both the patients’ cardiorespiratory stability and their pain intensity. The highest reward was given when pain was absent and both heart rate and respiration rate were within the acceptable range. By comparing the RL policy with physicians’ choices, Lopez-Martinez et al [41] found that RL policy tended to prescribe higher doses of morphine. This result was consistent with previous studies: continuous dosing provided similar or even better pain relief with no increase in acute adverse effects [70,71].

## Discussion

### Principal Findings

Our comprehensive review of the literature demonstrates that RL has the potential to be a clinical decision support tool in the ICU. As the RL algorithm is well aligned with sequential decision making in ICUs, RL consistently outperformed physicians in simulated studies. Nonetheless, challenges regarding RL system design, evaluation metrics, and model choice exist. In addition, all current applications have focused on using retrospective data sets to derive treatment algorithms and require prospective validation in authentic clinical settings.

### RL System Design

The majority of applications were similar in their formulation of the RL system design. The state space is usually constructed by features including patient demographics, laboratory test values, and vital signs, whereas some studies applied encoding methods to represent the state of the patients instead of using raw features. The action space was very specific to each application. For instance, in terms of the dosing category, the action space would be discretized ranges of medication dosage. For other categories, such as timing of an intervention, the action space would be the binary indicator of an intervention for each time step. The number of action levels differed among the studies. For some studies, the action levels could be as many as a dozen or a hundred (eg, optimal medication combination), whereas for other studies, the action levels were limited to only 2 (eg, on/off MV). The design of the reward function is central to successful RL learning. Most of the reward functions were designed a priori with guidance from clinical practice and protocols, but 2 studies [40,45] managed to directly learn the reward function from the data using inverse RL.

### Evaluation Metrics

The only metric that matters is if the adoption of an RL algorithm leads to improvement in some clinical outcomes. Most studies calculated the estimated mortality as the long-term outcome and drew plots to show the relationship between the estimated mortality versus the learnt value of patients’ state-action trajectories, where the higher value function was associated with lower mortality. The RL agent would provide treatment suggestions for those actions with higher values, thus leading to a lower estimated mortality. Estimated mortality is a popular metric for RL policy evaluation. However, the problem with the estimated mortality is that it is calculated from simulated trajectories with observational data, and may not be the actual mortality.

Mortality is not always the most relevant and appropriate outcome measure. For instance, in the study by Weng et al [46], they tried to identify individualized targeted blood glucose levels as a reference for physicians. In their study, 90-day mortality was used to evaluate the RL policy. However, a more relevant measure could be considered, such as short-term changes in the blood glucose level, physiological stability, and development of complications.

Several studies that focused on propofol titration have considered BIS as the evaluation metric to monitor the sedation level and hence to determine the effect of propofol. Although BIS monitoring is fairly objective, assessing sedation is usually performed by health care providers with clinically validated behavioral assessment scales such as the Richmond Agitation-Sedation Scale score [72]. In addition, EEG-based technologies, such as BIS and M-entropy, have been validated more in the operating room than in the ICU [73]. Furthermore, BIS cannot be used as the sole monitoring parameter for sedation, as it is affected by several other factors, including the anesthetic drugs used, muscle movement, or artifacts from surgical equipments [74].

To date, there has been no prospective evaluation of an RL algorithm. Moreover, the observational data itself may not truly reflect the underlying condition of patients. This is known as the partially observable MDP [75] problem, where we are only able to represent a patient's state by the observed physiological features, which are solved by mathematical approximation.

### Model Choice

FQI and DQN seem to be the top RL approaches among the reviewed studies. FQI is not a deep learning–based RL model, which guarantees convergence for many commonly used regressors, including kernel-based methods and decision trees. On the other hand, DQN leverages the representational power of DNNs to learn optimal treatment recommendations, mapping the patient state-action pair to the value function. Neural networks hold an advantage over tree-based methods in iterative settings in that it is possible to simply update the network weights at each iteration, rather than rebuilding the trees entirely.

Both FQI and DQN are off-policy RL models. Off-policy refers to learning about one way of behaving from the data generated by another way of selecting actions [76]. For instance, an off-policy RL model tries to train a policy X to select actions in each step, but it estimates the Q-values from state-action pairs where the action was chosen by following another policy Y. In contrast to off-policy learning, on-policy learning uses the same policy X to choose actions and to evaluate the returns in each step during training. Most of the included studies adopted off-policy RL models because the RL models aim to learn policy X from the data, which was generated by following real actions of physicians (policy Y). The data generated by policy Y is the actual physicians’ policy, where the RL models try to learn and improve from. This is the fundamental idea of applying off-policy RL models.

In addition, both FQI and DQN are value-based RL models that aim to learn the value functions. In value-based RL, a policy can be derived by following the action with the highest value at each time step. Another type of RL is called policy-based RL, which aims to learn the policy directly without worrying about the value function. Policy-based methods are more useful in continuous space. When the data volume is insufficient to train a DQN model, the DQN is not guaranteed to achieve a stable RL policy. As there is an infinite number of actions or states to estimate the values for, value-based RL models are too computationally expensive in the continuous space. However, policy-based RL models demand more data samples for training. Otherwise, the learned policy is not guaranteed to converge to an optimal one. Both value-based and policy-based RL models can be grouped in a more general way as *model-free* RL. Here the word *model-free* means the environment is unknown to an agent. The RL agent makes use of the trajectories generated from the environment, rather than explicitly knowing the rule or the transition probability. In contrast to model-free RL, *model-based* RL requires the agent to know the transition probability for all the state-action combinations explicitly and hence impractical as the state space and action space grow. In the critical care context, patients’ conditions and prognosis are very complex to apply model-based RL because we are not exactly sure about the probability of all state transitions. In addition, most studies in critical care could only use limited retrospective data to train the model offline. Therefore, we found that most of the studies have applied a value-based RL model to utilize the available observational data.

### Common Data Sets

We found that 71% (15/21) of applications utilized the MIMIC II or MIMIC III database to conduct their experiments. We conjecture that such popularity might be due to public availability and high quality of MIMIC data. However, data collected from a single source may introduce potential bias to the research findings. There are inherent biases in the medical data sets obtained at various institutions due to multiple factors, including operation strategy, hospital protocol, instrument difference, and patient preference. Therefore, the RL models trained on a single data set, regardless of the data volume, cannot be confidently applied to another data set. The findings from the reviewed articles may not be generalizable to other institutions and populations. In addition to the MIMIC database, one of the studies also utilized the eICU Research Institute (eRI) database to test their RL model [77]. The eRI database has a larger volume of data compared with the MIMIC database, and it is also publicly available. We suggest that future applications could cross-validate their models on both the MIMIC and eRI databases. In addition, all current applications have focused on using retrospective data sets to derive treatment algorithms and require prospective validation in authentic clinical settings.

### Strengths and Limitations of This Study

The strengths of this paper include the comprehensive and extensive search for all available publications that applied RL as an approach in the critical care context. Nonetheless, we acknowledge the limitations. We included papers (eg, those on arXiv) that have not been peer-reviewed *before* publication but these papers have undergone a postpublication peer review. According to the search phrases applied in this review, we may miss out certain papers that applied RL in critical care, but did not specify the phrase *intensive care* nor *ICU* in their full text papers.

### Challenges and Future Directions

A number of challenges must be overcome before RL can be implemented in a clinical setting. First, it is important to have a meaningful reward design. The RL agent would be vulnerable in case of reward misspecification, and might not be able to produce any meaningful treatment suggestion. Inverse RL can be an alternative to a priori–specified reward functions. However, inverse RL assumes that the given data represent the experts’ demonstrations and the recommendations from the data were already optimal; these may not be true.

Second, medical domains present special challenges with respect to data acquisition, analysis, interpretation, and presentation of these data in a clinically relevant and usable format. Addressing the question of censoring in suboptimal historical data and explicitly correcting for the bias that arises from the timing of interventions or dosing of medication is crucial to fair evaluation of learnt policies.

Third, another challenge for applying the RL model in the clinical setting is exploration. Unlike other domains such as game playing, where one can repeat the experiments as many times, in the clinical setting, the RL agent has to learn from a limited set of data and intervention variations that were collected offline. Using trial and error to explore all possible scenarios may conflict with medical ethics, thereby limiting the ability of the RL agent to attempt new behaviors to discover ones with higher rewards and better long-term outcomes.

In comparison with other machine learning approaches, there is an absence of acceptable performance standards in RL. This problem is not unique to RL but seems harder to address in RL compared with other machine learning approaches, such as prediction and classification algorithms, where accuracy and precision recall are more straightforward to implement. However, it is worth noting that RL has a distinct advantage over other machine learning approaches, that one can choose which outcome to optimize by specifying the reward function. This provides an opportunity to involve patient preferences and shared decision making. This becomes more relevant when learned policies change depending on the reward function. For example, an RL algorithm that optimizes survival may recommend a different set of treatments versus an RL algorithm that optimizes neurologic outcome. In such situations, patient preference is elicited to guide the choice of the RL algorithm.

RL has the potential to offer considerable advantages in supporting the decision making of physicians. However, certain key issues need to be addressed, such as clinical implementation, ethics, and medico-legal limitations in health care delivery [78]. In fact, any machine learning model would need to address these limitations carefully to serve as truly effective tools. In clinical practice, the RL models need to be refined iteratively throughout the time to include newly generated data from electronic health systems in hospitals, and the model must produce robust results for physicians to interpret and understand. Besides, patients’ understanding and willingness to use the RL model as a supporting tool in their care would be another important consideration. Another important ethical consideration would be the liability in case of medical error when the RL model recommendation differs from the physician. It has an impact on the autonomy of both the physician and patient. The problem of medical error works in both ways when there is a poor outcome: (1) if the physician follows the RL model recommendation, can the clinician then blame the model and the personnel who maintain the model; (2) if the clinician does not follow the RL model recommendation, can the clinician then be said to have made the wrong decision and be penalized.

Possible directions for future work include (1) modeling the RL environment as a partially observable MDP, in which observations from the data are mapped to some state space that truly represents patients’ underlying well-being; (2) extending the action space to be continuous, suggesting more precise and practical treatment recommendations to physicians; and (3) improving the interpretability of the RL models so that physicians can have more confidence in accepting the model results. With further efforts to tackle these challenges, RL methods could play a crucial role in helping to inform patient-specific decisions in critical care.

### Conclusions

In this comprehensive review, we synthesized data from 21 articles on the use of RL to process or analyze retrospective data from ICU patients. With the improvement of data collection and advancement in reinforcement learning technologies, we see great potential in RL-based decision support systems to optimize treatment recommendations for critical care.

## Appendix

Multimedia Appendix 1 Introduction to reinforcement learning.

Multimedia Appendix 2 Summary of study characteristics.

## Abbreviations

AI: artificial intelligence
aPTT: activated partial thromboplastin time
BIS: bispectral index
BUN: blood urea nitrogen
DNN: deep neural network
DQN: deep Q network
eRI: eICU Research Institute
FQI: fitted-Q-Iteration
ICU: intensive care unit
IV: intravenous
MAP: mean arterial pressure
MDP: Markov decision process
MIMIC III: Medical Information Mart for Intensive Care III
MV: mechanical ventilation
RCT: randomized controlled trial
RL: reinforcement learning
WBC: white blood cell
