# Supplementary material for: Reinforcement Learning for Clinical Decision Support in Critical Care: Comprehensive Review
Source: J Med Internet Res. 2020 Jul 20;22(7):e18477. doi: 10.2196/18477 (PMC7400046; doi:10.2196/18477)
Supplement: Multimedia Appendix 1 [file jmir_v22i7e18477_app1.pdf]

Supplementary File

Table of Contents

AN INTRODUCTION TO REINFORCEMENT LEARNING 2

POLICY-BASED REINFORCEMENT LEARNING 4

POLICY GRADIENT 4

VALUE-BASED REINFORCEMENT LEARNING 5

Q-LEARNING 5
DQN AND ITS DERIVATIVES 5
POLICY ITERATION AND VALUE ITERATION (FITTED Q ITERATION) 6
ACTOR-CRITIC REINFORCEMENT LEARNING 6

MODEL-BASED REINFORCEMENT LEARNING 6

INVERSE REINFORCEMENT LEARNING 7

REFERENCES 8

An Introduction to Reinforcement Learning

Reinforcement learning (RL) is one of the three main types of machine learning, where we may be more familiar with the other two types: supervised learning and unsupervised learning. Regressions and classifications are common supervised learning tasks and has been widely applied in the healthcare domain. Supervised Learning is task-driven, because there are always data labels to oversee or "supervise" the learning process. In contrast, unsupervised learning is data-driven where the learning process totally depends on the inter- or intra-relations among data clusters. Unlike supervised/unsupervised learning that learns the patterns or relations from data directly, RL tries to understand the data by interacting with the environment where the data came from. It is a goal-oriented learning algorithm wherein an agent or a decision maker perform a task by taking some actions in the environment and get evaluative feedbacks on the actions in each step, allowing it to improve the performance of subsequent actions.

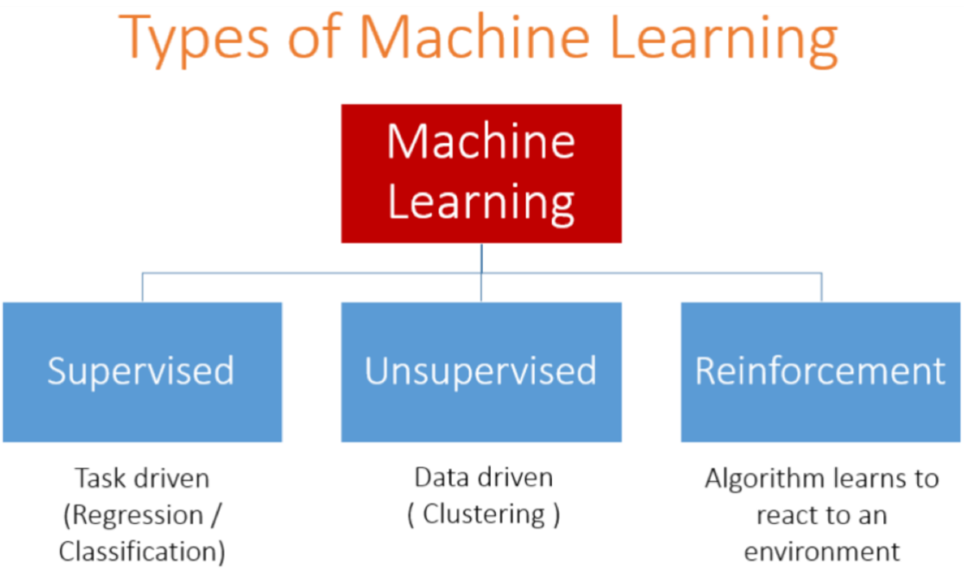

RL has been applied in various types of applications, such as robots movement, Atari game, autonomous vehicle, recommendation systems, financial investment etc. All the applications share the common goal: to select the best actions that maximise total reward for all the steps when interacting with an environment.

To achieve this goal, we need to take note of the unique characteristics in RL:

1. Actions may have long term consequences
2. Reward may be delayed
3. It may be better to sacrifice immediate reward to gain more long-term reward

With a chess game as an example, the very early moves for blocking the opponent might help winning the game in the end.

All the RL applications have the same sets of "building blocks", including

- **States**: Features space representing the environment, s_t
- **Actions**: A set of actions the agent can take, a_t
- **Reward**: The gain/loss of taking an action on a given state, $r_t = r(s_t, a_t)$
- **Transition Function**: It tells the agent what the next state would be if taking certain action at the current state. $P(s_{t+1}|\, s_t, a_t)$

- **Policy**: A mapping of state and action space, $\pi(a|s)$. The learning goal for RL can be understood as finding a policy, i.e. a mapping from state to action that can maximize accumulative reward: $\mathrm{E}[\sum_{t=0}^{\infty} r(s_t, a_t, s_{t+1})] = \mathrm{E}[\sum_{t=0}^{\infty} r_t]$

Mathematically, RL can be described by a Markov Decision Process (MDP) as shown in the following figure.

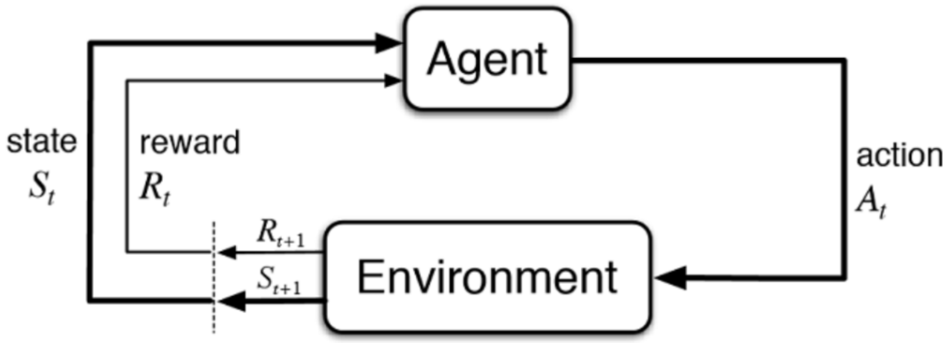

In a setting of a grid-world game (environment), the agent will need to walk from the start position to the goal position through the white grids. We design the rules as follows:

- Reward: -1 per time-step (the agent need to take the shortest path to the goal position to avoid negative reward)
- Actions: North, East, South, West in each step
- States: Agent's location in the grid-world

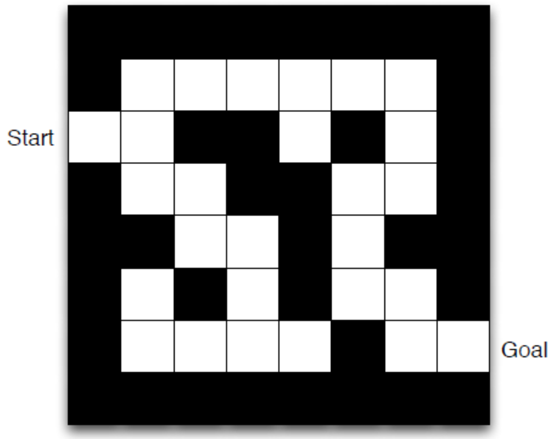

The agent would be able to conquer the game in either of the two ways:

1) The agent knows which direction to take in each of the white grid. In another word, the agent have a **policy function, $\pi(s)$** that tells the direction to move (actions) for all the states (shown as the red arrows).

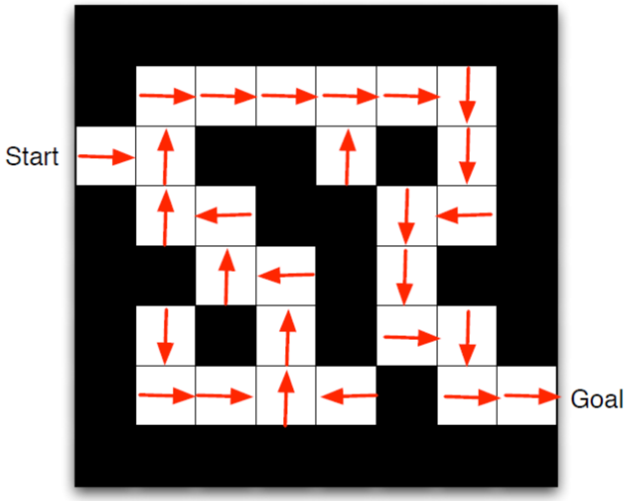

2) The agent knows the **value function, $\mathbf{V(s)}$** in each white grid from which the agent could compare the values for all adjacent states and take the action that leads to a state with a larger value in the subsequent step. The value function evaluate the goodness of states, and it could be written in another form with the action term: $\mathbf{V(s)} = \boldsymbol{E_{a\in A}[Q(s,a)]}$**.** The value function for a state s, is the average value for all the possible state-action pairs in that state. The value of the state-action pair $\boldsymbol{Q(s,a)}$ is called as **Q-function**.

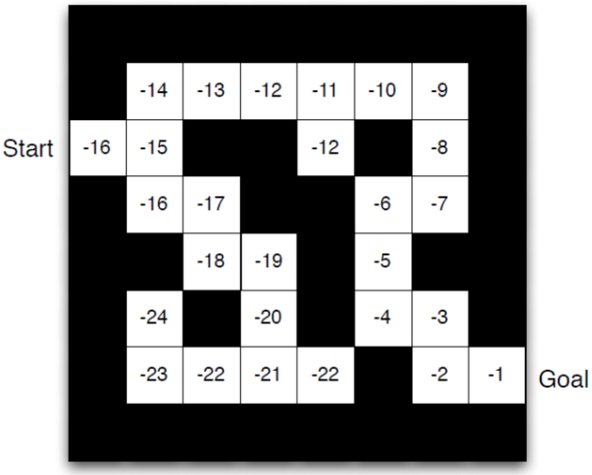

To summarise, an agent could solve an RL problem by either learning a policy function or a value/Q-function. The RL algorithms that learn the policy function is called **policy-based RL**, whereas those learn the value/Q-function is called **value-based RL**.

In the next section, we would introduction some common types of policy-based RL, value-based RL and some other extensions.

Policy-based Reinforcement Learning

Policy Gradient

The policy gradient[1] methods target at modelling and optimizing the policy directly. The policy $\pi_\theta(a|s)$ is usually modelled by a neural network with parameter θ. When an agent act according to a policy π_θ from a random initial state s_1, the policy will tell the agent to choose

action a_1 under the state s_1. The environment would respond to this action with a reward r_1, and would lead the agent to enter the next state s_2. The state-action sequence would continue to roll out to form a trajectory τ, with reward at each step by following the policy π_θ. The objective of the agent is to continuously update the policy π_θ, so that the average accumulated reward $\overline{R_\theta}$ from the trajectory τ could be maximized. $\overline{R_\theta} = E_{\tau \sim p_\theta(\tau)}[R(\tau)]$

The policy could be updated by adjusting the parameter θ by taking gradient to the reward $\overline{R_\theta}$ in the neural network. Therefore, this type of RL is named policy gradient.

Value-based Reinforcement Learning

Q-learning

The main idea for Q-learning[2] is to construct a reference map of values and state-action pairs, so that given a random initial state s_1, an agent would refer to the reference map to seek action a_1 which maximize the value for state s_1, $Q(s_1, a_1)$. Q-function could be re-write in terms of the reward: $Q(s_t, a_t) = r(s_t, a_t) + \gamma \max_{a_{t+1}} Q(s_{t+1}, a_{t+1})$, where the value of the current state-action pair $Q(s_t, a_t)$ is defined as the reward $r(s_t, a_t)$ received from the current action plus the estimate from the highest Q-value of the next possible state-action pair with a discount factor $\gamma \max_{a_{t+1}} Q(s_{t+1}, a_{t+1})$. The discount factor γ has the value between 0 to 1 and it was used to represent the decay effect of future reward w.r.t. time. The reward from near future would have a greater impact than the reward from the future that is further away.

The reference map for state-action values can be construct using a table, named Q-table, where each entry represents the value of one state-action pair. However, as the state/action space grow in size, the number of entries in Q-table would have to grow geometrically to store all the values, which makes Q-learning not feasible for problems with continuous state/action space.

DQN and its Derivatives

Deep Q Network (DQN)[3] is a derivative of Q-learning, where the Q-table is replaced by a deep neural network (DNN) parametrized by θ, to represent the value of the state-action pairs. The rule for updating parameter θ is to minimize the mean squared error (loss function) given as

$$L(\theta) = 0.5 \left\| Q_\theta(s_t, a_t) - r(s_t, a_t) + \gamma \max_{a_{t+1}} Q_\theta(s_{t+1}, a_{t+1}) \right\|^2$$

Here the DQN algorithm suffer from a limitation of overestimation of Q-function, because both Q-value $Q_\theta(s_t, a_t)$ and the action term $\max_{a_{t+1}} Q_\theta(s_{t+1}, a_{t+1})$ was chosen from the same network modelled by θ. To mitigate this issue, Hasselt et al. designed a model which could estimate the Q-value and the action separately with two DQN network, **Double DQN**[4]. Therefore the loss function of Double DQN becomes:

$$L(\theta_1, \theta_2) = 0.5 \left\| Q_{\theta 1}(s_t, a_t) - r(s_t, a_t) + \gamma \max_{a_{t+1}} Q_{\theta 2}(s_{t+1}, a_{t+1}) \right\|^2$$

where θ_1, θ_2 refers to the network parameters for the two DQN respectively.

Another modification on Double DQN is to separate the Q-value with two steams, value stream and advantage steam, $Q(s_t, a_t) = V(s_t) - A(s_t, a_t)$. The network is called **Double DQN**

with Dueling[5]. The key motivation behind this architecture is that for some cases, it is unnecessary to know the value of each action at every timestep. By explicitly separating two estimators, the dueling architecture can learn which states are (or are not) valuable, without having to learn the effect of each action for each state.

Policy Iteration and Value Iteration (Fitted Q iteration)

Policy Iteration [6] update a policy in 3 steps. The first step is the initialization of a random policy π. The second component is policy evaluation. By policy evaluation, we mean that following this policy, what should be the value of any state. As mentioned above, given a policy π, the value of a state is the expected reward when the agent starts from s and follows π after that. $V(s_t) = \sum_{s_{t+1},r} p^{\pi}(s_{t+1}, r|s_t)\,[r(s_t, a_t) + \gamma\, V(s_{t+1})$, where $p^{\pi}(s_{t+1}, r|s_t)$ is the probability of entering state s_{t+1} with reward r by following the policy π from state s_t. The third component is policy improvement. Policy π will be updated to π' if the new policy produce a higher value of $V(s_t)$. We run policy evaluation and improvement iteratively until the policy becomes stable when none of the action maximization step in any state causes a change in the policy.

Value Iteration[7] or **Fitted Q Iteration**[8] follows exactly the same steps as Policy Iteration. The only difference is to replace the policy π with an estimation with a value function, $\pi = argmax_a\, Q(s, a)$.

Actor-Critic Reinforcement Learning

Actor-Critic RL [9] is a combination of policy-based RL and value-based RL. It has two networks which are parameterized with DNN. One is called actor-network and the other one called critic-network. The critic-network is similar as those value-based RL, where the network estimate the value function or the Q-function. The actor-network updates policy as those in the policy-based RL, where it improve the policy in the suggested by the critic-network. Actor-Critic RL has two main advantages over pure policy-based and valued based RL. 1) Convergence is guaranteed even for non-linear approximation of the value function (which is not the case for Q-learning). 2) Actor-Critic RL reduce variance with respect to pure policy search methods.

Model-based Reinforcement Learning

All the above discussed RL algorithms are all model-free RL, in which we assume the transition function $P(s_{t+1}|\, s_t, a_t)$ is unknown. Therefore, given the current state and action pair, the RL agent won't be able to tell what the real next state is. In fact, the model-free RL does not attempt to learn the transition function explicitly. It bypasses the transition function by sampling from the environment. While in **model-based RL**[10], the agent aims to learns the transition function from the environment, so that given the current state and an action, an model-based RL algorithm would estimate the probability of all possible next states. With model-based RL, one can generate new samples from an environment easily.

In the model-based RL, we first act in the environment to collect a few trajectories of state-action pairs. Then we deduce a model with DNN or Monte Carlo Tree Search. With this model, we would be able to generate new trajectories. In the next step, we update the value function or the policy function from the generated trajectories, and use the updated value function/ policy function to go back to the environment to select action. This process repeats over and

over again to gradually improve the model so that it fully represents the feedbacks from the real environment.

Inverse Reinforcement Learning

In most RL algorithms, the reward function is hand-crafted without knowing the true reward. This type of reward design is very vulnerable to misspecification. **Inverse RL**[11] can be the alternative to the hand designed reward function where the Inverse RL learns the reward directly through expert demonstrations. The Inverse RL aims to learn an optimal policies from sub-optimal demonstrations. The goal of Inverse RL is to recover the right reward function.

The general idea behind Inverse RL with sampled trajectories is to iteratively improve a reward function by comparing the value of the approximately optimal expert policy with a set of generated policies. Here are a few key steps in Inverse RL.

1. Estimate the value of our optimal policy for the initial state $\hat{V}^{\pi}(s_1)$, as well as the value of every generated policy $\hat{V}^{\pi_i}(s_1)$, by taking the average cumulative reward of many randomly sampled trajectories.
2. Generate an estimate of the reward function R by solving a linear programming problem. Specifically, set α_i in reward function $R(s) = \alpha_1\emptyset_1(s) + \alpha_2\emptyset_2(s) + \cdots + \alpha_d\emptyset_d(s)$,to maximize the difference between our optimal policy and each of the other k generated policies.
3. Repeat step 1 and 2 multiple iterations and add the newly generated policy the set of k candidate policies, and repeat the procedures.

Another way to learn the reward function is through DNN, where the input to the DNN are the state-action pairs (trajectories) produced by a sub-optimal policy , and the output of the DNN is a reward $R_{\varphi}(s,a)$, where φ is the parameters of the DNN that we would learn through backpropagation.

References

1. Sutton, R.S., et al. *Policy gradient methods for reinforcement learning with function approximation*. in *Advances in neural information processing systems*. 2000.
2. Watkins, C.J. and P. Dayan, *Q-learning.* Machine learning, 1992. **8**(3-4): p. 279-292.
3. Mnih, V., et al., *Human-level control through deep reinforcement learning.* Nature, 2015. **518**(7540): p. 529-533.
4. Van Hasselt, H., A. Guez, and D. Silver. *Deep reinforcement learning with double q-learning*. in *Thirtieth AAAI conference on artificial intelligence*. 2016.
5. Wang, Z., et al., *Dueling network architectures for deep reinforcement learning.* arXiv preprint arXiv:1511.06581, 2015.
6. Lagoudakis, M.G. and R. Parr, *Least-squares policy iteration.* Journal of machine learning research, 2003. **4**(Dec): p. 1107-1149.
7. Tamar, A., et al. *Value iteration networks*. in *Advances in Neural Information Processing Systems*. 2016.
8. Riedmiller, M. *Neural fitted Q iteration–first experiences with a data efficient neural reinforcement learning method*. in *European Conference on Machine Learning*. 2005. Springer.
9. Grondman, I., et al., *A survey of actor-critic reinforcement learning: Standard and natural policy gradients.* IEEE Transactions on Systems, Man, and Cybernetics, Part C (Applications and Reviews), 2012. **42**(6): p. 1291-1307.
10. Doya, K., et al., *Multiple model-based reinforcement learning.* Neural computation, 2002. **14**(6): p. 1347-1369.
11. Ng, A.Y. and S.J. Russell. *Algorithms for inverse reinforcement learning*. in *Icml*. 2000.
