# Supplementary material for: Reinforcement Learning for Clinical Decision Support in Critical Care: Comprehensive Review
Source: J Med Internet Res. 2020 Jul 20;22(7):e18477. doi: 10.2196/18477 (PMC7400046; doi:10.2196/18477)
Supplement: Multimedia Appendix 2 [file jmir_v22i7e18477_app2.pdf]

Table 2. Summary of study characteristics.

Reference	Category	Purpose	Disease/symptom	Number of patients	Main method	Evaluation	Relevant outcome	Data source
Weng et al, 2017 [37]	Optimal laboratory value	To find personalized target laboratory values as references for clinical decision making	Sepsis	5565	Policy iteration	(1) Computed the empirically estimated mortality rate of the real glycemic trajectory; (2) Plot expected return with respect to mortality rate	(1) Learned optimal policy could reduce the patients' estimated 90-day mortality rate by 6.3%, from 31% to 24.7%; (2) Plot showed a negative correlation	MIMIC[a] III
Wang et al, 2018 [38]	Medication choice	To find the time-varying medications according to the dynamic states of patients	NA[b]	22,865	Actor-critic network with LSTM[c]	(1) Plot estimated in-hospital mortality rates versus expected return; (2) Estimate the hospital mortality by following RL[d] policy	(1) The plot of estimated mortality showed inverse relationship with regard to expected return; (2) Estimated hospital mortality was reduced by 4.4% by following the RL policy compared with hospital policy	MIMIC III

Prasad et al, 2017 [34]	Optimal timing; optimal dosing of a medication	To find the best timing of on/off invasive MV[e]; to infuse the optimal dosing of sedation (propofol)	Patients in ICUs[f] who need to be supported with invasive MV.	2464	FQI[g] with tree regressor and neural network as regressor	Calculate the degree of consistency between learnt FQI policy and hospital policy in terms of MV setting and dosing of propofol	For the timing of MV, the learnt RL policy matched hospital policy in 85% of transitions. For dosing of propofol, RL policy achieved 58% accuracy	MIMIC III
Cheng et al, 2019 [35]	Optimal timing	To find optimal timing of order the 4 laboratory test (WBC[h], creatinine, lactate, and blood urea nitrogen)	Sepsis or acute renal failure	6060	FQI with multiobjective Gaussian process	(1) Reduction in SOFA[i] score; (2) Treatment onset after taking a laboratory test; (3) Laboratory redundancy (information gain); (4) Absolute laboratory cost	(1) The RL agent outperforms the hospital policy across all reward components; (2) Time intervals for treatment is higher in hospital policy than RL policy across all 4 laboratory tests; (3) The mean information in laboratory tests ordered by physicians is consistently outperformed by RL policy: for WBC, 44% reduction; for lactate, 27% reduction in number of orders	MIMIC III

Yu et al, 2019 [36]	Optimal timing; optimal dosing of a medication	To find the best timing of on/off invasive MV; to infuse the optimal dosing of sedation (propofol)	Patients in ICUs who need to be supported with invasive MV	707	FQI with Bayesian inverse RL	(1) Calculate percentage of consistency of FQI policy and hospital policy; (2) Rank feature importance	(1) RL policy matches 53.5% of the joint action of physicians, with 99.6% consistency in ventilation action and 53.9% in sedative action; (2) Feature importance showed that physicians pay more attention to patients' physiological stability (eg, heart rate and respiration rate), rather than oxygenation criteria (FiO2[j], PEEP[k], and SpO2[l])	MIMIC III
Borera et al, 2011 [20]	Optimal dosing of a medication	To find optimal dosing of sedation (propofol) for a target BIS[m] value	NA	1000	Q-learning with neural network	Calculate the median performance error, the median absolute performance error, and the root mean square error	The median performance error was 0.02%, the median absolute performance error was 0.84% and the root mean square error was 3.8 BIS for the RL agent	Simulated data

Padmanabhan et al, 2015 [21]	Optimal dosing of a medication	To find dosing of sedation (propofol) while maintaining the MAP^{n} value	NA	30	Epsilon-greedy policy iteration	Evaluate the target values of the BIS and MAP using median performance error , median absolute performance error, and root mean square error.	The median performance error, median absolute performance error, root mean square error for BIS were 3.97%, 4.19% and 2.12-3.30 respectively; The median performance error, median absolute performance error, and root mean square error for MAP were 4.05%, 5.31% and 2.30-9.50	Simulated data
Padmanabhan et al, 2017 [22]	Optimal dosing of a medication	To achieve target BIS value while adjusting dosing of sedation (propofol and remifentanil)	NA	25	Q-learning	Calculate mean performance error , the median performance error, and median absolute performance error	For propofol and remifentanil the mean performance error was 0.61%, the median performance error was 0.11% and the median absolute performance error was 0.27%	Simulated data
Padmanabhan et al, 2019 [23]	Optimal dosing of a medication	To achieve target BIS value while adjusting dosing	NA	10	Actor-critic network with prespecified pharmacological	Plot the BIS value with respect to time for different	Patients with different states show that the proposed RL agent can achieve robustness to	Simulated data

		of sedation (propofol)			math model of a simulated patient	infusion rate of propofol over time	pharmacological parameters differences and provide an optimal dosing of propofol	
Nemati et al, 2016 [6]	Optimal dosing of a medication	To find optimal dosing of unfractionated heparin	Patients who received a heparin intravenous infusion during their ICU stay	4470	FQI with neural network	Plot average reward versus discrepancy between the RL agent policy and hospital policy	From the plot, on average and consistently over time, following the recommendations of the RL agent results in the best long-term performance (accumulated reward)	MIMIC II
Ghassemi et al, 2018 [24]	Optimal dosing of a medication	To find optimal dosing of unfractionated heparin	Patients who received a heparin intravenous infusion	4470	Policy gradient RL	Accuracy and AUC[o] for the RL agent compared with the hospital policy	Accuracy for the RL agent was 58%, and AUC was 0.73	MIMIC III
Lin et al, 2018 [25]	Optimal dosing of a medication	To find optimal dosing of unfractionated heparin	Patients admitted to ICUs with the need for infusion of heparin	2598; 2310	Actor-critic network	(1) Plot average reward versus discrepancy between the RL agent policy and hospital policy; (2) Regression over treatment and	(1) When there is no discrepancy between RL agent and hospital, the reward is highest from the plot; (2) RL policy has significant association with anticoagulant complications (*P*<.05)	MIMIC III; Emory Health

						complication outcome		
Raghu et al, 2017 [27]	Optimal dosing of a medication	To find the optimal dosing of intravenous fluid and vasopressors	Sepsis	17,898	Double DQN[p] with dueling	Plot observed mortality versus the difference between the dosages recommended by RL agent and hospital	The plot shows a V-shape curve, whereas mortality is lowest when there is no discrepancy between RL agent and hospital policy	MIMIC III
Raghu et al, 2017 [26]	Optimal dosing of a medication	To find the optimal dosing of intravenous fluid and vasopressors	Sepsis	17,898	Double DQN with dueling	(1) Compute empirically derived function of proportion of mortality versus expected return; (2) Compute the mean discounted return of chosen actions under the hospital policy	(1) Estimated mortality was 13.9 % for hospital policy and it is improved up to 4% if follow RL agent policy; (2) Expected return for physician was 9.87 and this value was increased to 10.73 for RL agent	MIMIC III

Raghu et al, 2018 [28]	Optimal dosing of a medication	To find the optimal dosing of intravenous fluid and vasopressors	Sepsis	17,898	Model-based RL	Policy evaluation includes the use of the (1) Per-horizon weighted Importance sampling, (2) Per-horizon weighted doubly robust, and (3) Approximate model estimators	Highest value for per-horizon weighted Importance sampling and per-horizon weighted doubly robust was 12.1 and 12.8 respectively when following hospital policy in low and high SOFA group, and follow RL agent policy in medium SOFA group; Highest value for approximate model was 9.36 when following hospital policy in low, medium and high SOFA groups	MIMIC III
Komorowski et al, 2018 [15]	Optimal dosing of a medication	To find the optimal dosing of intravenous fluid and vasopressors	Sepsis	17,083; 79,073	Policy Iteration	Plot mortality versus dosing discrepancy between RL agent and hospital policy	From the plot, the patients who received the treatments suggested by the AI[q] clinician had the lowest mortality rate	MIMIC III; eRI
Futoma et al, 2018 [29]	Optimal dosing of a medication	To find the optimal dosing of intravenous	Sepsis	9255	Multioutput Gaussian process	(1) Plot estimated mortality versus value of RL policy;	(1) The mortality-value plot showed a negative association; (2) The	Duke University Hospital

		fluid, vasopressors, and antibiotics			deep recurrent Q-networks	(2) Plot estimated mortality versus discrepancy between the dosages recommended by RL agent and hospital	mortality-discrepancy plot showed a V-shape curve, where the mortality is lowest when there is no discrepancy between the RL policy and the hospital policy	
Peng et al, 2018 [30]	Optimal dosing of a medication	To find the optimal dosing of intravenous fluid and vasopressors	Sepsis	15,415	DQN with kernel method	Off-policy evaluation via the weighted doubly robust estimator	The DQN with kernel model has a weighted doubly robust value of 5.72 which outperformed the physician's value of 3.76	MIMIC III
Lee et al, 2019 [31]	Optimal dosing of a medication	To find the optimal dosing of vasopressors	Sepsis	17,898	Inverse RL	(1) Plot the learnt reward with respect to patient's vitals; (2) Calculate the proportion of the RL-recommended action in the physicians' actions for each discrete	(1) The IRL[r] model places higher rewards on high vasopressor for patients with low platelet counts, low blood pressure and high heart rate and no vasopressor is preferred when the platelet counts, blood pressure and heart rate are stable; (2) The recommend action for	MIMIC III

						dosage bin of vasopressors	dosing range of vasopressors match on average 80% of those actions by clinicians in the data	
Petersen et al, 2018 [33]	Optimal dosing of cytokine	To find the optimal dosing of cytokine	Sepsis	500	Deep deterministic policy gradient	Calculate the mortality rate for the simulated patients from the IIRABM[s] close-loop system	The learned treatment strategy was showed to achieve 0.8% mortality over 500 randomly selected patient parameterizations with mortalities average of 49%	Simulated patients with IIRABM
Lopez-Martinez et al, 2019 [32]	Optimal dosing of a medication	To find the optimal dosing of morphine	Pain	6843	Double DQN with dueling	Plotting the RL agent actions versus physician's actions	The actions recommended from the model for the 94.2% instances in which physicians chose to withhold morphine	MIMIC III

[a]MIMIC: Medical Information Mart for Intensive Care.

[b]NA: not applicable

[c]LSTM: long short-term memory.

[d]RL: reinforcement learning.

[e]MV: mechanical ventilation.

[f]ICU: intensive care unit.

[g]FQI: fitted-Q-iteration.

[h]WBC: white blood cell.

[i]SOFA: sequential organ failure assessment

[j]FiO2: fraction of inspired oxygen

[k]PEEP: positive end-expiratory pressure

[l] SpO2: oxygen saturation

[m]BIS: bispectral index.

[n]MAP: mean arterial pressure.

[o]AUC: area under the receiver operating characteristic curve

[p]DQN: deep Q network.

[q]AI: artificial intelligence

[r]IRL: inverse reinforcement learning

[t]IIRABM: innate immune response agent-based model.
